# Systemic Sclerosis and Silicone Breast Implant: A Case Report and Review of the Literature

**DOI:** 10.1155/2014/809629

**Published:** 2014-09-30

**Authors:** Antonios Psarras, Ioannis Gkougkourelas, Konstantinos Tselios, Alexandros Sarantopoulos, Panagiota Boura

**Affiliations:** Clinical Immunology Unit, 2nd Department of Internal Medicine, Hippokration General Hospital, Aristotle University of Thessaloniki, Konstantinoupoleos Street 49, 546 42 Thessaloniki, Greece

## Abstract

Environmentally induced systemic sclerosis is a well-recognized condition, which is correlated with exposure to various chemical compounds or drugs. However, development of scleroderma-like disease after exposure to silicone has always been a controversial issue and, over time, it has triggered spirited debate whether there is a certain association or not. Herein, we report the case of a 35-year-old female who developed Raynaud's phenomenon and, finally, systemic sclerosis shortly after silicone breast implantation surgery.

## 1. Introduction

Systemic sclerosis is a connective tissue disease, which is characterized by endothelial dysfunction, immunologic activity, and fibrosis. Systemic sclerosis is classified further into diffuse cutaneous systemic sclerosis and limited cutaneous systemic sclerosis according to the extent and distribution of skin involvement and immunologic findings. As far as the pathogenesis of the disease is concerned, repeated vascular injury is thought to trigger immune responses in predisposed individuals. Immune responses involving both innate and adaptive immunity result to fibroblast activation and finally, to extensive fibrosis of visceral organs and microangiopathy [1]. Even though the exposure to various chemical compounds, such as silica and other organic solvents, has been correlated with the pathogenesis of the disease, the definite correlation between systemic sclerosis and silicone implants has frequently been doubted [2, 3].

## 2. Case Presentation

The patient was admitted in November 2012, 4 months after operation, complaining about Raynaud's phenomenon. In addition, during the following months she developed thickening of the fingers (puffy fingers) and symptoms of gastroesophageal reflux disease. Then, nailfold capillaroscopy was compatible with active scleroderma pattern and esophagogastroduodenoscopy showed lower esophageal sphincter dysfunction and grade 2 esophagitis. High resolution computed tomography (HRCT) of the thorax revealed bilateral basal ground glass opacities and nonpathological hilar lymphadenopathy (<1 cm), while there was no evidence that the silicone breast implant had ruptured or leaked. Diffusing capacity of the lung for carbon monoxide (DLCO) was slightly impaired. Finally, there was no evidence of pulmonary hypertension in heart ultrasound (sPAP: 22 mmHg).

Immunological screening showed high titres of antinuclear antibodies (1 : 5120), but other autoantibodies (anti-Scl-70, anticentromere, and anti-U1-RNP) were negative. Erythrocyte sedimentation rate (ESR) was normal and C3/C4d complement fragments were slightly reduced.

The diagnosis of systemic sclerosis was definite according to the 2013 ACR/EULAR criteria for classification of systemic sclerosis and the patient was put on treatment with methotrexate, nifedipine, and pantoprazole [4]. The follow-up of the patient did not reveal any significant changes regarding lung disease or other complications, since HRCT was operated again 12 months after initial diagnosis and DLCO had no further reduction. Therapy with proton pump inhibitors (PPIs) seemed to be beneficial for gastroesophageal reflux disease symptoms. Complete blood count and ESR remained normal, but immunological screening showed persistent high titres of antinuclear antibodies.

## 3. Discussion

Although many cases have been referred to in the literature since early 1980s, the correlation between silicone breast implants and connective tissue diseases, such as systemic sclerosis, systemic lupus erythematosus, Sjögren's syndrome, or localized skin conditions like morphea, is yet to be proven [5–7]. Many case-control studies were designed in the previous decades to investigate a possible association, but all of them do not support this hypothesis. The literature has been reviewed by searching in databases (PubMed) relevant articles published until June 2014 with the following keywords: systemic sclerosis; scleroderma; silicone; breast implant. The results of the most relevant case-control studies and meta-analyses are summarized in Table 1.

A large case-control study by Englert and Brooks included women in Sydney, Australia, who had been diagnosed with scleroderma during 1974–1988. Additionally, control patients were selected randomly from 29 Sydney general practices [8]. That study included 251 scleroderma cases and 289 controls. The rate of silicone-augmentation mammoplasty in scleroderma patients was 1,59% in comparison with 1,73% in control patients and the study failed to demonstrate an association between silicone breast implantation and the subsequent development of systemic sclerosis. Data from the Sydney study were reanalyzed to validate self-reported augmentation mammoplasty status in 556 scleroderma patients and 289 general practice controls [9]. Results verified again that silicone breast implantation was not an environmental inducer of systemic sclerosis. Another case-control study by Burns et al. reported in 1996 was conducted in Michigan, USA. 274 women diagnosed with systemic sclerosis during 1985–1991 and 1184 controls were recruited. Breast implants did not seem to increase the risk for development of systemic sclerosis [10].

Furthermore, a case-control study by Goldman et al. recruited 4229 female patients from only one rheumatology practice in Atlanta, USA, during 1986–1991. 721 patients were diagnosed with a connective tissue disease, including 64 with systemic sclerosis. There was no history of silicone breast implantation in any of the 64 patients with systemic sclerosis [11].

Results from case-control study by Hochberg et al. failed to demonstrate an important connection between silicone and systemic sclerosis. The study included 837 women with a clinical diagnosis of the disease and 2507 controls. Only 11 patients (1,31%) with systemic sclerosis underwent a breast implant surgery in the past in comparison with 31 (1,24%) of the controls [12].

A large Danish follow-up study included 2761 women with breast implants and 8807 women underwent a breast reduction operation to investigate the linkage between silicone and connective tissue diseases [13]. After a mean follow-up duration of 11,5 years (public clinics) and 6,8 years (private clinics) there was no evidence that silicone implant surgery increases the incidence of connective tissue diseases, including systemic sclerosis, among females.

A meta-analysis for scleroderma and silicone breast implants taking under consideration three case-control studies (Burns et al., Englert et al., and Hochberg et al.) did not support the hypothesis that women with silicone-augmentation mammoplasty are at higher risk for developing systemic sclerosis [14]. In addition, two other meta-analyses by Janowsky et al. and Perkins et al. showed no correlation [15, 16]. A recent prospective study which assessed the connection between systemic sclerosis and occupational exposure to chemical compounds, including silicone implants, showed no association [17].

Several studies have tried to find a link between silicone and autoimmunity. It has been suggested that silicone breast implants might act as foreign bodies that trigger immune responses, which may lead to the production of several autoantibodies, such as anti-silicone antibodies [18]. Moreover, fibroblast proliferation can be triggered by macrophages exposed to silicone and it might be linked to interstitial lung disease [19]. In contrast, results from silicone administration in tight skin (TSK/+) mice come to oppose any supported association [20].

## 4. Conclusions

This case indicates another case of systemic sclerosis that might be associated with silicone breast implant. Currently, recent epidemiologic investigations and meta-analyses generally conclude that silicone breast implantation cannot cause connective tissue disease, but multicentral collaborations are needed to conclude more definite results about the incidence of similar cases. However, when autoimmunity occurs, the question about the causative factor is always open.

## Figures and Tables

**Table 1:** Results of different studies for correlation between systemic sclerosis and silicone breast implants.

Case-control studies	Patients	Controls	Correlation
Englert et al. [9]	251	289	No
Burns et al. [10]	274	1184	No
Goldman et al. [11]	721	3508	No
Hochberg et al. [12]	837	2507	No

Follow-up study	Correlation		

Kjøller et al. [13]	No		

Meta-analyses	Correlation		

Whorton and Wong [14]	No		
Janowsky et al. [15]	No		
Perkins et al. [16]	No		
